# Fungal community structure of fallen pine and oak wood at different stages of decomposition in the Qinling Mountains, China

**DOI:** 10.1038/s41598-017-14425-6

**Published:** 2017-10-24

**Authors:** Jie Yuan, Xiaofeng Zheng, Fei Cheng, Xian Zhu, Lin Hou, Jingxia Li, Shuoxin Zhang

**Affiliations:** 1 College of Forestry, Northwest A&F University, Yangling, Shaanxi 712100 China; 2 Qinling National Forest Ecosystem Research Station, Huoditang, Ningshan, Shaanxi 711600 China; 3 College of Science, Northwest A&F University, Yangling, Shaanxi 712100 China; 4 Guangxi University, Forestry College, Nanning, Guangxi 530004 China; 5 Gansu Forestry Technological College, Tianshui, Gansu 741020 China

## Abstract

Historically, intense forest hazards have resulted in an increase in the quantity of fallen wood in the Qinling Mountains. Fallen wood has a decisive influence on the nutrient cycling, carbon budget and ecosystem biodiversity of forests, and fungi are essential for the decomposition of fallen wood. Moreover, decaying dead wood alters fungal communities. The development of high-throughput sequencing methods has facilitated the ongoing investigation of relevant molecular forest ecosystems with a focus on fungal communities. In this study, fallen wood and its associated fungal communities were compared at different stages of decomposition to evaluate relative species abundance and species diversity. The physical and chemical factors that alter fungal communities were also compared by performing correspondence analysis according to host tree species across all stages of decomposition. Tree species were the major source of differences in fungal community diversity at all decomposition stages, and fungal communities achieved the highest levels of diversity at the intermediate and late decomposition stages. Interactions between various physical and chemical factors and fungal communities shared the same regulatory mechanisms, and there was no tree species-specific influence. Improving our knowledge of wood-inhabiting fungal communities is crucial for forest ecosystem conservation.

## Introduction

The Qinling Mountains in central China are an important climate boundary between the southern subtropics and the northern temperate zone where the typical types of vegetation from both climate zones assemble together, resulting in astonishingly high biodiversity in a veritable plant “kingdom”^1^. However, in recent decades, the Huoditang Forest Region in the Qinling Mountains has been constantly affected by wind and pests, resulting in increased quantities of fallen wood of two major species: *Pinus tabulaeformis* and *Quercus aliena* var. *acuteserrata*. Fallen wood is defined as downed or leaning deadwood (>45° from the vertical) with a minimum diameter ≥10 cm at the widest point and a length ≥1 m^2,3^. Fallen wood plays an important role in the forest ecosystem by providing wildlife habitats, facilitating nutrient cycling, and supplying carbon and nitrogen sources for micro-communities^4–6^. Fallen wood is also the substrate for multicellular forest species, acts as a seedbed and is a key component of forest biodiversity^7–10^.

The ecological function of fallen wood is primarily realized via the process of decomposition, during which fallen wood releases carbon, nitrogen, phosphorus and other nutrients^11,12^. Different trees undergo different wood decay processes. Decomposition is controlled by climatic factors as well as biome factors. Wood-decaying fungi primarily affect fallen wood decomposition at specific locations and are among the most common saproxylic organisms, comprising thousands of taxa^13^. Much of our knowledge of the process of wood decomposition in forest ecosystems involves wood-inhabiting fungi. Wood properties, including nutrients, lignin and stoichiometry, control or are controlled by fallen wood-inhabiting organisms^14–16^. To advance our understanding of modern forest ecosystems and the ecology of dead wood, it is essential to characterize the interaction between fallen wood and wood-inhabiting fungi.

Molecular studies focused on fungal communities associated with fallen wood at different stages of decomposition are being carried out with increasing frequency^17^. Modern molecular techniques improve our understanding of fallen wood decomposition. Sequence-based studies of DNA obtained directly from fallen wood have revolutionized our view of the wood-inhabiting fungal community^18^. The comparison of terminal restriction fragment length polymorphisms (RFLPs) targeting the internal transcribed spacer (ITS) regions of rRNA genes with high-throughput sequencing methods enables detailed, semiquantitative analysis of fungal communities in large sample nests and provides ecological information that extends far beyond terminal RFLPs in terms of both detail and magnitude^17^. Consequently, the use of high-throughput sequencing to study fungal communities increases accuracy and reliability.

Changes in wood structure and chemical composition during wood decomposition (primarily due to fungi) result in further changes to species composition and alter later decomposition processes. Fungal species differ during specific stages of decomposition, indicating that structural attributes are important when determining species assemblages, and fallen wood must be examined at different stages of decomposition to construct fungal assemblages^19^. Multilinear studies have examined fungal communities during the decomposition of fallen wood obtained from different trees, located in different regions, and demonstrating different levels of community diversity. For example, in a study of Norway spruce fallen wood in Sweden, fungal diversity tended to increase with sample decay stage^20^. Tree species diversity is an important factor in fungal species composition^21^, as species-rich communities exert negative impacts on the rate of decomposition^22^. However, species richness increases the decay rate due to different resource requirements and activity patterns among fungal species, as well as different environmental conditions^23^. There is still no consensus regarding the factors that impact fungal community diversity, and only a few publications have investigated differences in decay-associated fungi on different fallen wood hosts.

Hence, our objectives in this study were (i) to study the fungal species diversity and relative abundance of *P*. *tabulaeformis* and *Q*. *aliena* var. *acuteserrata* and (ii) to compare the physical and chemical factors within these two different fallen wood host species that affect the wood-inhabiting fungal community during decomposition. Knowledge of wood-inhabiting fungal communities, which also control the wood decay process, is important for both forest ecosystem conservation and to understand fungal community patterns within decaying dead wood.

## Results

### Overview of sequence assignments

A total of 371,130 sequencing reads were obtained from all samples, and we analysed 339,716 optimized, useful reads (Table 1). The reads obtained from *P*. *tabulaeformis* samples accounted for 159,221 of the total reads, with an average sequence length of 301.32 bp, while the 180,495 reads obtained from *Q*. *aliena* var. *acuteserrata* samples had an average sequence length of 301.686 bp.

**Table 1:** The summary of the optimized reads.

Sample	Read	Base (bp)	Average length (bp)
RCL-I	49095	13609629	277.21
RCL-II	22400	7064407	315.38
RCL-III	34086	11301971	331.57
RCL-IV	41913	11782967	281.13
RCL-V	33001	10004002	303.14
YS-I	21439	6202118	288.29
YS-II	36151	10905120	301.65
YS-III	44721	13449605	300.74
YS-IV	30414	9473631	310.49
YS-V	26496	8039801	303.43

### Fungal community structure during decomposition succession

Fungal community members at different taxonomic levels and different stages of decomposition for two studied tree species are summarized in Table 2. These data provide an overview of the fungal community structures of these two species. We obtained 94–298 operational taxonomic units (OTUs) from *Q*. *aliena* var. *acuteserrata* samples and 125-263 OTUs from *P*. *tabulaeformis* samples at the species level. *P*. *tabulaeformis* samples exhibited higher fungal species diversity than *Q*. *aliena* var. *acuteserrata* samples at the family, genus and species levels. Thus, *P*. *tabulaeformis* supports a more diverse fungal community than *Q*. *aliena* var. *acuteserrata*.

**Table 2:** Summary of fungal group numbers at different taxonomy levels.

Sample	Phylum	Class	Order	Family	Genus	Species
RCL-I	6	13	20	24	31	147
RCL-II	6	14	20	34	43	201
RCL-III	4	10	12	15	23	94
RCL-IV	6	11	18	19	20	154
RCL-V	6	15	24	33	41	298
YS-I	6	13	24	36	46	263
YS-II	5	9	12	14	21	125
YS-III	8	18	25	35	42	260
YS-IV	6	12	18	26	29	233
YS-V	4	10	17	29	33	260
Total	9	23	47	73	139	324

**Figure 1:**
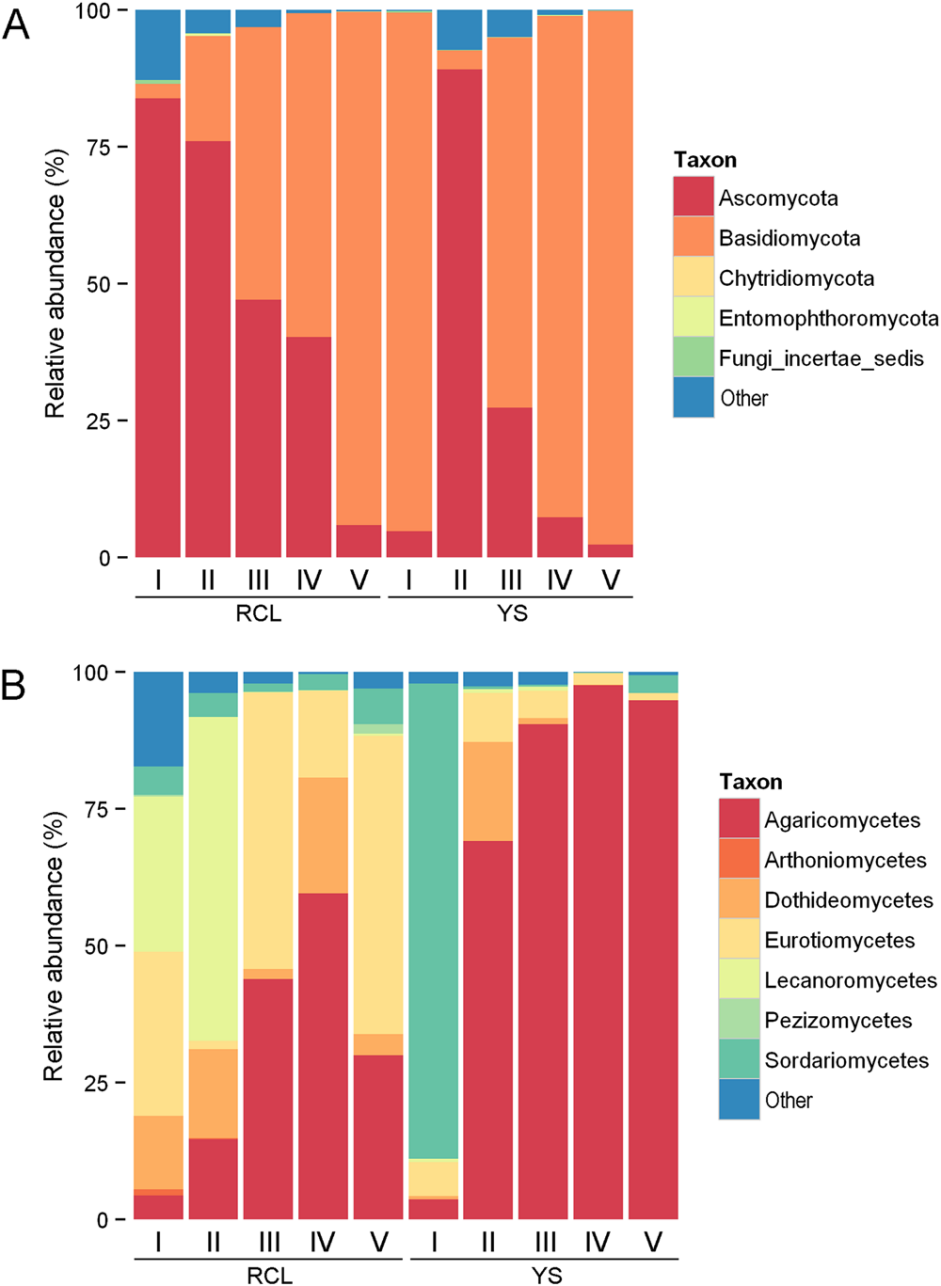
Variation in relative abundance of fungal phylums (**A**) and classes (**B**) at different decomposition stages.

The majority of the fungal communities derived from both the *P*. *tabulaeformis* and *Q*. *aliena* var. *acuteserrata* samples consisted of the phyla Basidiomycota and Ascomycota (Fig. 1A). Fungal communities were categorized by class (Fig. 1B), and the relative abundance of each fungal community differed (Fig. 1). In *Q*. *aliena* var. *acuteserrata* samples, 5 fungal classes were observed: Agaricomycetes (41.72%), Eurotiomycetes (30.13%), Dothideomycetes (12.34%), Lecanoromycetes (8.28%) and Sordariomycetes (3.73%). In *P*. *tabulaeformis* samples, 3 classes were observed: Agaricomycetes (74.80%), Sordariomycetes (18.91%) and Eurotiomycetes (3.37%).

**Figure 2:**
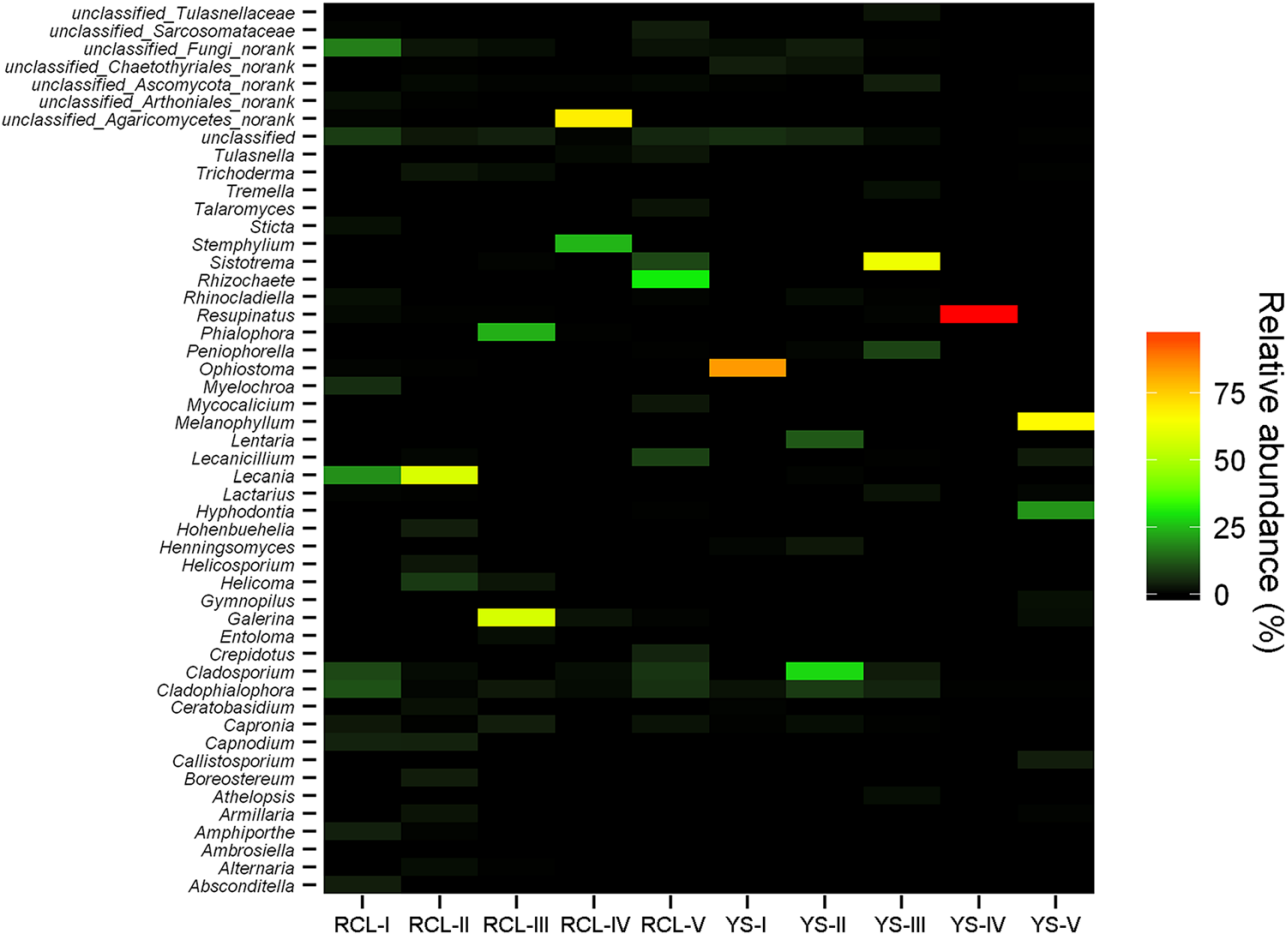
Relative abundance heatmap for fungal genus at different decomposition stages.

Fungal community composition heat maps at different stages of decomposition for these two wood species are displayed in Fig. 2, revealing relative abundance at the genus level. Most of Fig. 2 is coloured black or green. A few bars are colour-coded orange to red to indicate their abundance levels. Seven samples each contained a dominant fungal genus with a relative abundance of over 50%. For *Q*. *aliena* var. *acuteserrata* samples, the relative abundances of both the *Lecania* genus and the *Galerina* genus were 58% in RCL-II. An unclassified Agaricomycetes-no rank genus with a relative abundance of 69% was identified in RCL-IV samples. Among *P*. *tabulaeformis* samples, the *Resupinatus* genus had a relative abundance of 99% in YS-IV, the *Ophiostoma* genus had a relative abundance of 84% in YS-I, the *Melanophyllum* genus had a relative abundance of 67% in YS-V and the *Sistotrema* genus had a relative abundance of 63% in YS-III.

**Figure 3:**
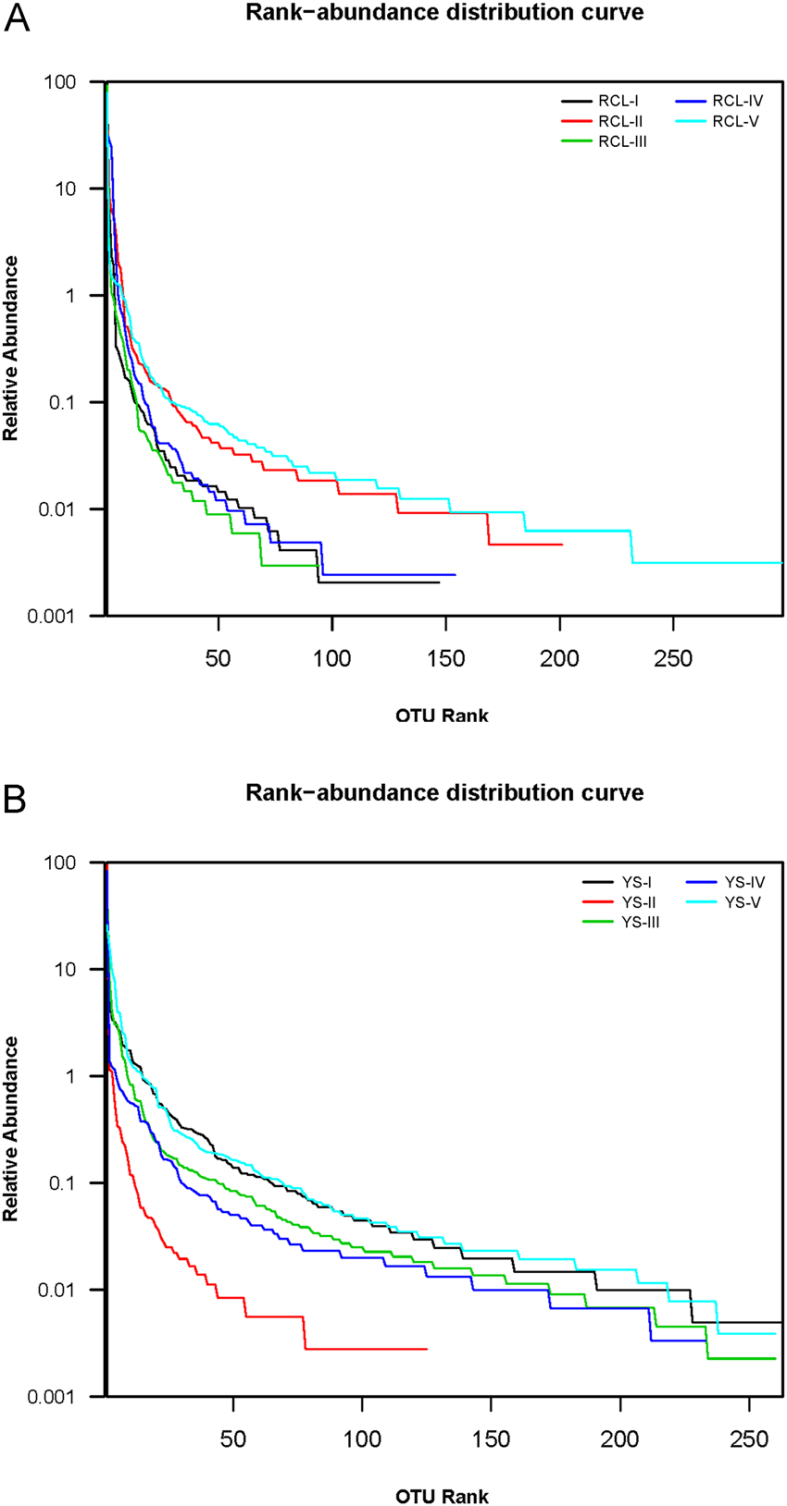
Rank-Abundance curves of fungal OTUs of *Q*. *aliena* var. *acuteserrata* (**A**) and *P*. *tabulaeformis* (**B**) at different decomposition stages.

The rank abundance curves for OTUs from all samples provided relative abundance levels and visualized both fungal species richness and evenness (Fig. 3). Certain *Q*. *aliena* var. *acuteserrata* samples, specifically RCL-I, RCL-III and RCL-IV, contained fewer fungal species and exhibited low species evenness, particularly RCL-III. The others, RCL-II and RCL-V, had similar slopes for their best-fit lines, with the exception of the length of the X-axis for RCL-V. The two RCL-V samples contained more fungal species and demonstrated higher species evenness. The *P*. *tabulaeformis* samples exclusive to YS-II contained many fungal species types, similar to RCL-V, and exhibited similar high relative abundance, although RCL-V had the highest abundance among all samples.

**Figure 4:**
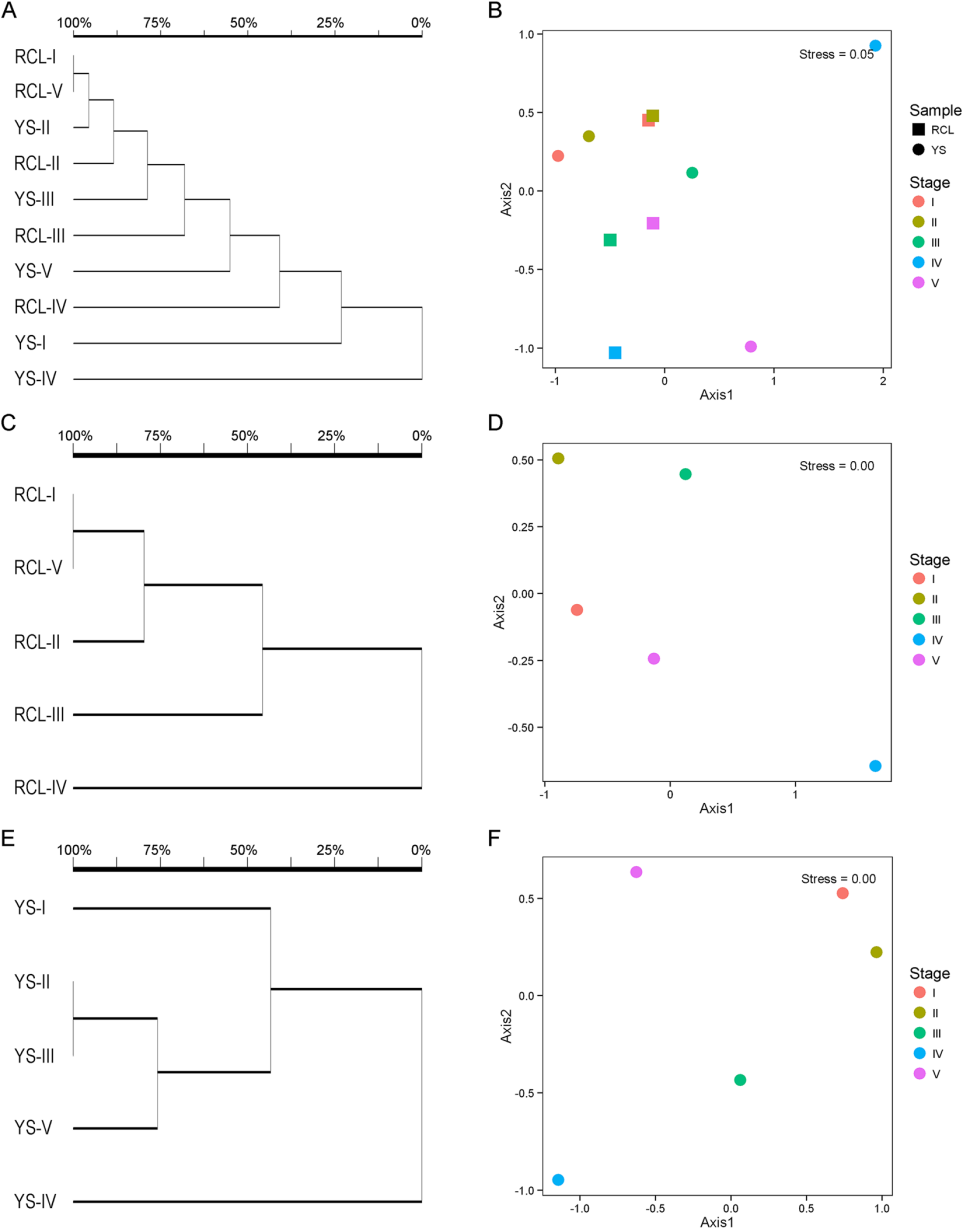
Clustering (left) and NMDS (right) analyses of fungal communities in fallen woods at different decomposition stages at the genus level.

Cluster analysis based on Euclidean similarity distance, together with non-metric multidimensional scaling (NMDS) based on Bray-Curtis similarity distance, revealed similarities in species composition at the genus level (Fig. 4). The NMDS stress value (0.05) indicates excellent ordination representation. Fungal community composition significantly differed with the progression of decomposition. The largest differences in fungal community composition for both *P*. *tabulaeformis* and *Q*. *aliena* var. *acuteserrata* samples appeared during decomposition stage IV and then narrowed somewhat compared with the composition during initial state I. Nevertheless, the difference in fungal community composition for *P*. *tabulaeformis* narrowed only slightly between decomposition stage IV and V, unlike *Q*. *aliena* var. *acuteserrata*. Furthermore, both tree species exhibited a high degree of similarity in terms of fungal community composition during decomposition stages I and III and high discrepancy levels during stages IV and V.

### Correspondence analysis

**Figure 5:**
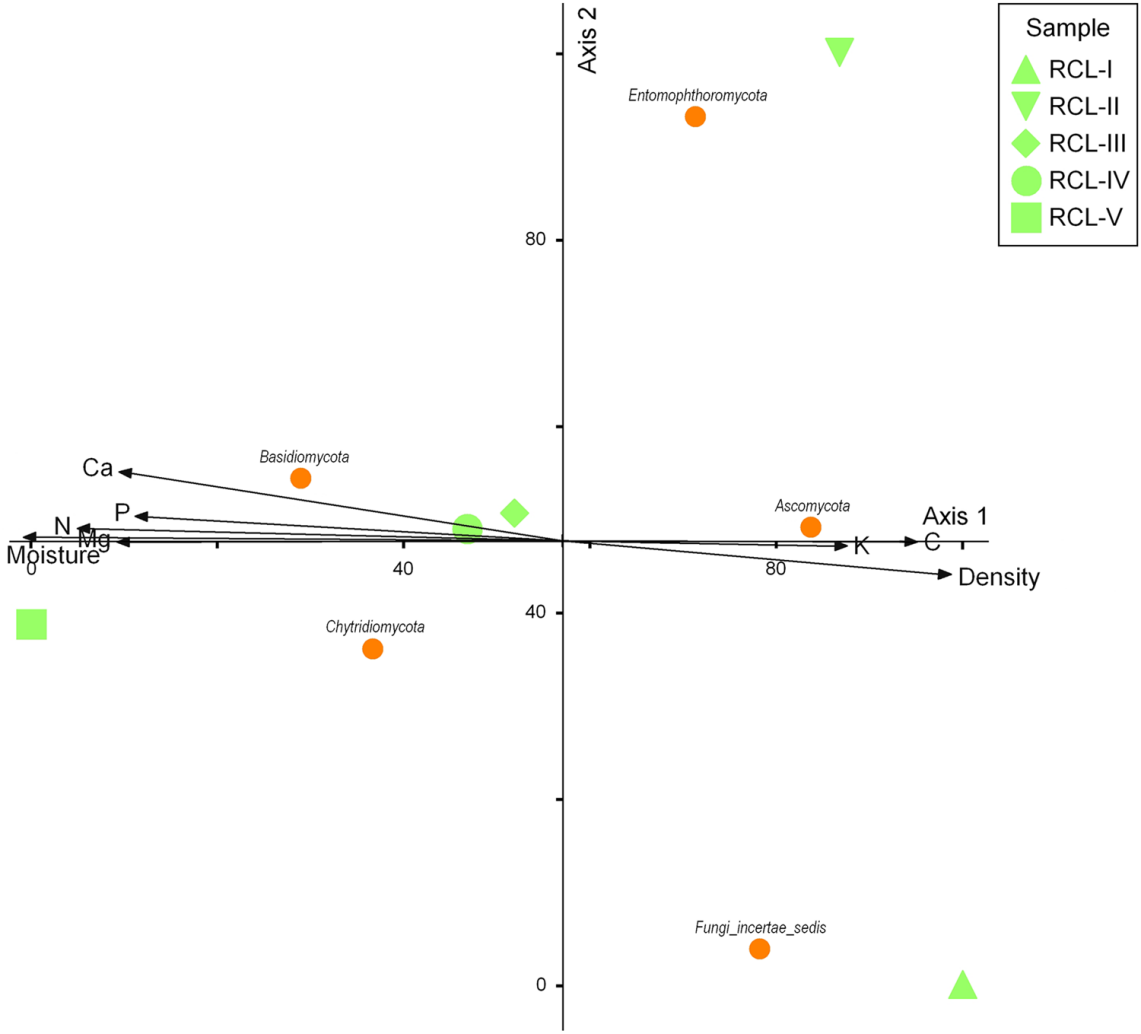
Correspondence analysis of physical and chemical factors and fungal communities of *Q*. *aliena* var. *acuteserrata*.

**Figure 6:**
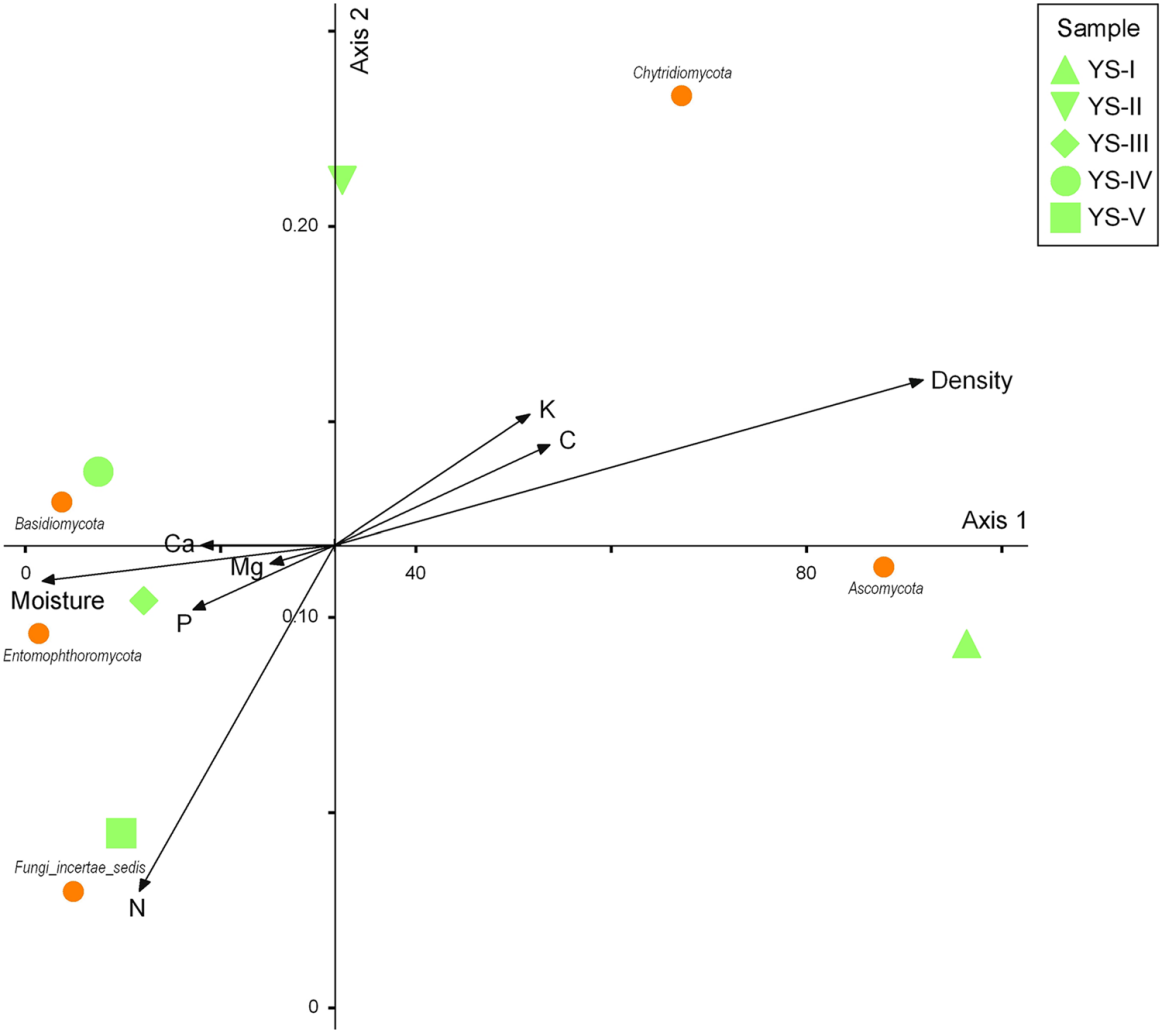
Correspondence analysis of physical and chemical factors and fungal communities of *P*. *tabulaeformis*.

Correlations between the physical and chemical parameters of each fallen wood species and fungal community were discerned from a correspondence analysis, as shown in Figs 5 and 6, respectively. The length of a physical or chemical parameter arrow indicates the strength of that parameter with regard to the overall fungal community. The influence of each physical or chemical parameter on *Q*. *aliena* var. *acuteserrata* was approximate.

Density and moisture appeared to be the most important physical and chemical parameters. Carbon, potassium, phosphorus, magnesium, calcium and nitrogen were the secondary impact factors. For *P*. *tabulaeformis*, density and nitrogen were the most powerful physical and chemical parameters, while the influence of minor factors, i.e., carbon, potassium, phosphorus, magnesium, calcium and moisture, on the fungal community was relatively weak compared with their influence in the *Q*. *aliena* var. *acuteserrata* fungal community.

## Discussion

**Figure 7:**
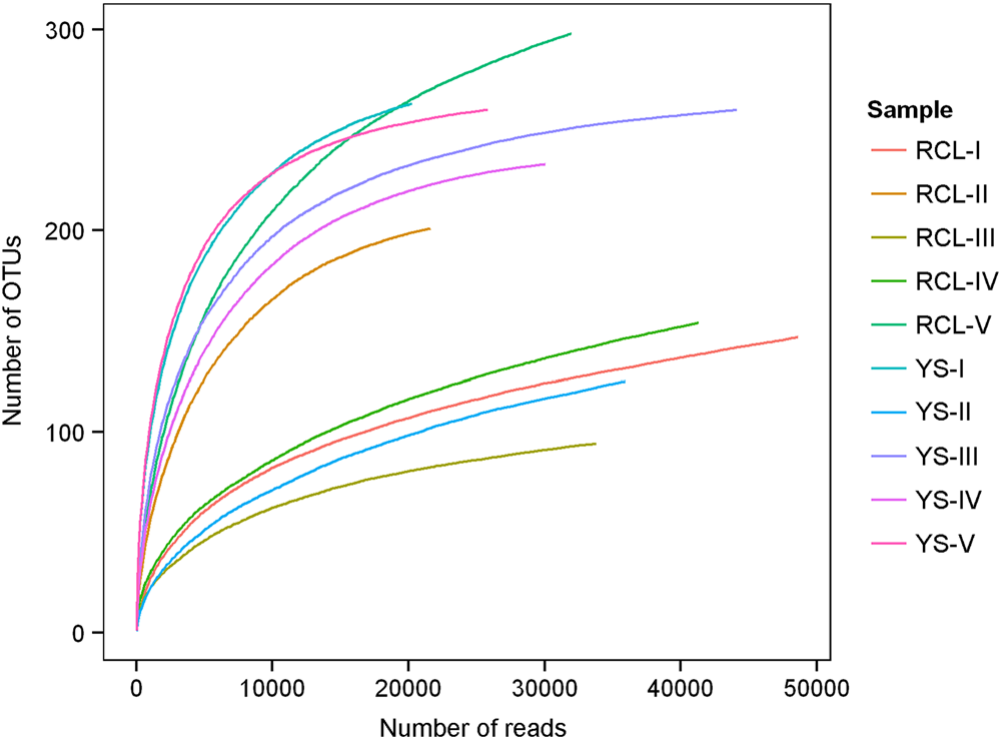
Rarefaction curves of fungal OTUs, designated as OTU_0.03_.

We obtained sufficient sequencing outputs for the pine species *P*. *tabulaeformis* and the oak species *Q*. *aliena* var. *acuteserrata*. The rarefaction curves of fungal OTUs were plotted to test the reliability of the sequence outputs and to predict the maximum number of OTUs at a similarity level of 97% (Fig. 7). Some of the rarefaction curves plotted based on these results failed to reach saturation, but coverage estimators using Chao1^24,25^ reflected the enormous diversity found in fallen wood samples. These samplings, RCL-II, YS-I and YS-V, initially stabilized their curves at 20,000 reads. When the number of reads reached 40,000, the curve of each sample became steady, indicating that the sequencing results were adequate and reasonable.

Fungal richness obviously increased during the final stage for *Q*. *aliena* var. *acuteserrata*, while only a slight increase occurred for *P*. *tabulaeformis*. Baldrian^26^ reported a slight increase during the final decomposition stage in temperate forests, which is similar to what we observed for *P*. *tabulaeformis* fallen wood. Fukasawa^27^ observed a peak fungal richness during the final stage in decayed fallen wood, which explains our findings in *Q*. *aliena* var. *acuteserrata*. We assume that the significant differences between the substrates provided by *P*. *tabulaeformis* and *Q*. *aliena* var. *acuteserrata* explain these differences in species richness, although further studies are necessary to investigate this issue.

Direct sequencing of fallen wood samples elucidated the fungal community at different stages of decomposition. Fallen wood during various stages of decomposition provides habitats for a variety of organisms, including fungal communities^28^. The phyla Basidiomycota and Ascomycota are the most frequently encountered wood-inhabiting fungi^29,30^ in fallen wood. Classes Agaricomycete, Sordariomycetes and Eurotiomycetes were all observed in both *P*. *tabulaeformis* and *Q*. *aliena* var. *acuteserrata* fallen wood. Arslanova suggested the existence of fungal communities in both coniferous and broad-leafed species^31^ in her dissertation. Decaying pine wood during all decomposition stages exhibits high diversity among wood-inhabiting fungi^32^. In her dissertation, Arslanova mentioned the existence of the fungal community in both coniferous and broad-leafed species^31^. The fungal communities identified at the class level in both pine and oak fallen wood samples aligned with the results of Kirker^33^. The unclassified genus belonging to Agaricomycetes, observed in RCL-IV with an abundance of 69%, was unexpected but explainable because the morphology of Agaricomycetes demonstrates an unparalleled diversity^34^. A similar taxonomic distribution was present across all of the samples from both tree species at the phylum and class levels. Visible differences in the taxonomic distribution first attracted our attention at the genus level. A distinct separation was observed for the species diversity of dead wood fungi based on their host tree. Host tree species are known to delimit wood-inhabiting fungi. Bîrsan^35^ found that this separation occurred even among three coniferous host trees; additionally, Küffer^36^ verified the distinction between coniferous and broadleaf host tree species. Differences in fungal species abundance in our study were consistent with these patterns.

Decaying fallen wood provides a snapshot of the nutrients and organic substances available for wood-inhabiting microorganisms^37^. Fungi predominantly prefer middle and late decomposition succession stages^38^. We speculate that the lifestyle of the observed fungi is saprophytic, allowing them to invade heavily decayed wood. Wood decomposition represents a stabilizing force within dead wood^39^, and a new equilibrium is reached when decomposition reaches stage V. Changes such as decreased wood density, a decreased C/N ratio and increased mineral and nitrogen content develop during the decomposition succession of fallen wood. As density decreases, fungal richness peaks during the intermediate decomposition stages, such as decay classes III and IV^40^. A lower C/N ratio, together with higher mineral and nitrogen contents, in dead wood during late decomposition succession stages results in the availability of nitrogen sources for wood-inhabiting fungi, regardless of forest floor vegetation^41^. Thus, there are clear changes in fungal community diversity with the succession of decomposition.

Fungal communities from *Q*. *aliena* var. *acuteserrata* and *P*. *tabulaeformis* reflect physical and chemical factors. Correlations between physical and chemical factors are rarely tree species-specific^42^. Unique and mutual fungal OTUs derived from sequences play critical roles, and discrepancies in fungi explain the mildly different results. Density and moisture (precipitation) are factors that differ with different wood decomposition stages^43^ and exert the most significant effects. Analysis has indicated that nitrogen, phosphorus, and the C/N ratio are determining factors for fungal composition^44^. A meta-analysis of 36 studies from all forested continents revealed that nitrogen, phosphorus, and the C/N ratio correlate with the decomposition rates of angiosperms^45^. Wood decomposition results from the activities of decomposer organisms. The presence of fungal communities and their structure, dynamics, and diversity are explicit and susceptible to the status of decaying wood^46^. Thus, factors controlling the decomposition rates of angiosperms control the associated fungal communities.

This study focused on comparing fungal community diversity and richness during the decomposition of *P*. *tabulaeformis* and *Q*. *aliena* var. *acuteserrata* fallen wood. Furthermore, we analysed correlations among the fungal community, the physical and chemical factors impacting *P*. *tabulaeformis* and *Q*. *aliena* var. *acuteserrata* fallen wood and the stages of decomposition. Differing tree species were the major source of differences in fungal community structure during all stages of decomposition, and the fungal community reached its highest diversity levels during the intermediate and late decomposition stages. The physical and chemical factors and fungal communities of *Q*. *aliena* var. *acuteserrata* and *P*. *tabulaeformis* shared the same regulatory mechanisms, and there were no tree species-specific influences. Thus, this comparative study provides sufficient evidence to understand the relationship between the decomposition of fallen wood and fungal community dynamics in natural forests and will aid the accurate prediction of shifts in fungal community composition and function according to the decomposition stage and tree species of fallen wood. In addition, our study elucidated physical and chemical factors influencing fungal community structure during different decomposition stages in fallen wood, providing a theoretical basis for the protection of fungal resources.

## Methodology

### Study site

The total study area of 2037 ha is located in the Huoditang Experimental Forest Farm of Northwest A&F University in the Qinling Mountains, Shaanxi Province, China. The climate belongs to a warm temperate zone, with a mean annual temperature ranging from 8-10 °C, annual precipitation ranging from 900–1200 mm, and a frost-free period of 170 days. The abrupt and broken topography mainly consists of granite and gneiss with a mean slope of 35°. The soil is classified as loam with a mean soil depth of 45 cm. Human activities in this region have largely disappeared since the natural forest protection project was initiated in 1998.

In the summer of 2013, we plotted *P*. *tabulaeformis* and *Q*. *aliena* var. *acuteserrata* forests into six sites. The plots were 60 m × 60 m rectangles with three replicates for both the *P*. *tabulaeformis* and *Q*. *aliena* var. *acuteserrata* forests. According to an investigation of fallen wood in each plot, most logs were e 20-30 cm in size. Thus, fallen wood with a diameter of 25 ± 5 cm was selected in each plot. Each plot was distributed on nearly flat terrain with similar site conditions. In the *P*. *tabulaeformis* forest, the altitude was 1484–1564 m, and the geographic coordinates were N33°25′11″−33°26′05″ and E108°26′21″−108°27′11″. The *P*. *tabulaeformis* forest was 60 years old and was dominated by *P*. *tabulaeformis* (75% of all trees), with a forest canopy density of 70%. The mean stand height, diameter at breast height (DBH) and stand density were 16 m, 24 cm and 1328 trees · ha^−1^, respectively. For the *Q*. *aliena* var. *acuteserrata* forest, the altitude was 1567-1621 m, and the geographic coordinates were N33°25′53″−33°26′21″ and E108°26′08″−108°26′21″. The *Q*. *aliena* var. *acuteserrata* forest was 50 years old and was dominated by *Q*. *aliena* var. *acuteserrata* (70% of all trees), with a forest canopy density of 75%. The mean stand height, DBH and stand density were 13 m, 18 cm and 1735 trees · ha^−1^, respectively.

### Sampling

We conducted sampling in July 2013. Samples of fallen wood were collected and processed in the same manner^47^ and were assigned to one of five decay classes based on discrepancies in internal and external tissue characteristics^48^ with sufficient (at least three) replicates. A total of 100 samples were collected and cut using a handsaw, while late decomposition succession samples were simply transplanted into a sterile aluminium box. Within each site, upper parts of the cross-section were preferred to obtain soil microorganisms. Each piece fallen wood was sampled at both ends and in the middle and shipped to the laboratory immediately. A −80 °C refrigerator was used to store the samples.

### DNA extraction and sequencing

Samples from the same pieces of fallen wood were mixed into one sample after liquid nitrogen grinding at a frequency of 30 Hz for 2 min. DNA was isolated using a PowerSoil^®^ DNA Isolation Kit (MO BIO Corp.) according to the manufacturer’s instructions. High-throughput sequencing was performed with a GeneAmp^®^ 9700 sequencer (ABI Corp.). Primers were designed based on the fungal *ITS1* region. The forward primer was 1737F (GGA AGT AAA AGT CGT AAC AAG G), and the reverse primer was 2043 R (GCT GCG TTC ATC GAT GC), both with inserted barcodes. Each 20-μl PCR reaction consisted of 4 μl of 5 × FastPfu Buffer, 2 μl of dNTPs (2.5 mM), 0.4 μl of forward primer (5 μM), 0.4 μl of reverse primer (5 μM), 0.4 μl of FastPfu Polymerase, 10 ng of template DNA and ddH_2_O. The following protocol was used for PCR: initial denaturation at 95 °C for 2 min, followed by 30 cycles of 95 °C for 30 s, 50 °C for 30 s, and 72 °C for 30 s, with a final extension at 72 °C for 5 min. PCR products were recovered using an AxyPrepDNA Gel Recovery Kit (Axygen Corp.) and were quantitatively detected with a QuantiFluor™-ST fluorometer (Promega Corp.). A MiSeq library was constructed based on the combination of appropriately proportioned DNA fragments and was sequenced (2 × 300 BP) on an Illumina platform.

### Data analysis

Biological statistical analysis of OTUs was conducted at the 97% similarity level using Usearch (version 7.1 http://qiime.org/)^49,50^. The RDP Bayesian algorithm was used to classify the representative sequences, and the community composition of each sample was statistically analysed at various levels (phylum, class, order, family, genus and species). Fungal ITS region sequences were identified based on reference sequences in the public database Unite (Release 5.0 http://unite.ut.ee/index.php). Taxonomy analysis was performed using Qiime^51^ (http://qiime.org/scripts/assign_taxonomy.html) and RDP Classifier^52^ (version 2.2 http://sourceforge.net/projects/rdp-classifier/). Mothur^53^ was used for diversity analysis, and cluster analysis and NMDS were conducted for community similarity analysis. Relative graphic progress was tracked using R i386 3.1.2. Correspondence analysis was performed to investigate the effects of physical and chemical factors on the fungal community at different stages of decomposition and was visualized using PC-ORD 5.0. *Q*. *aliena* var. *acuteserrata* was abbreviated to RCL, and *P*. *tabulaeformis* was abbreviated to YS, according to Mandarin pronunciations. I, II, III, IV and V represent different decomposition stages, i.e., I means the initial stage, while V means the final stage.

## Electronic supplementary material

Supplementary information
