# Supplementary material for: Fungal community structure of fallen pine and oak wood at different stages of decomposition in the Qinling Mountains, China
Source: Sci Rep. 2017 Oct 24;7:13866. doi: 10.1038/s41598-017-14425-6 (PMC5654975; doi:10.1038/s41598-017-14425-6)
Supplement: Supplementary file 1 — Supplementary information [file 41598_2017_14425_MOESM1_ESM.pdf]

Fungal community structure of fallen pine and oak wood at different stages of decomposition in the Qinling Mountains, China

Jie Yuan[a,1], Xiaofeng Zheng[a,1], Fei Cheng[a,d], Xian Zhu[a,c], Lin Hou[a,b], Jingxia Li [a,e], Shuoxin Zhang[a,b*]

[a]College of Forestry, Northwest A&F University, Yangling, Shaanxi 712100, China

[b]Qinling National Forest Ecosystem Research Station, Huoditang, Ningshan, Shaanxi 711600, China

[c]College of Science, Northwest A&F University, Yangling, Shaanxi 712100, China

[d]Guangxi University, Forestry College, Nanning, Guangxi 530004, China

[e]Gansu Forestry Technological College, Tianshui, Gansu 741020, China

[1] These authors contributed equally to this work.

*Corresponding author. E-mail: sxzhang@nwsuaf.edu.cn; Tel: +86-29-87082993; Fax: +86-29-87082993

Supplementary information

S1 Table. Characteristics of fallen woods at different decomposition stages in forest system.

S2 Figure. Moisture and density of fallen woods of *Q. aliena* var. *acuteserrata* and *P. tabulaeformis* at different decomposition stages.

S3 Figure. Chemical characteristics for fallen woods of *Q. aliena* var. *acuteserrata* and *P. tabulaeformis* at five decomposition stages.

S4 Figure. Number patterns of specific and shared fungal OTUs in fallen woods of *Q.*

aliena var. *acuteserrata* (A) and *P. tabulaeformis* (B) at different decomposition stages.

S1. Characteristics of fallen woods at different decomposition stages in forest system.

Characteristics	Decomposition stages				
	I	II	III	Ⅳ	V
Structure integrity	Sound	Sapwood slightly rotting, heartwood sound	Sapwood missing, heartwood mostly sound	Heartwood decayed	Soft
Leaves	Present	Absent	Absent	Absent	Absent
Branches	All twig present	Larger twig present	Larger branches present	Branch stubs present	Absent
Bark	Present	Present	Often present	Often absent	Absent
Bole shape	Round	Round	Round	Round to oval	Oval to flat
Wood consistency	Solid	Solid	Semisolid	Partly soft	Fragmented to powdery
Color of wood	Original color	Original color	Original color to faded	Original color to faded	Heavily faded
Position of log on ground	Elevated on support point	Elevated on support point	Near or on ground	All of log on ground	All of log on ground
Invaded by roots	No	No	Sapwood area	Throughout	Throughout
Indirect measure	Cambium still fresh, died less than 1 year	Cambium decayed, knife blade penetrates a few millimeters	Knife blade penetrates less than 2cm	Knife blade penetrates 2～5cm	Knife blade penetrates all the way

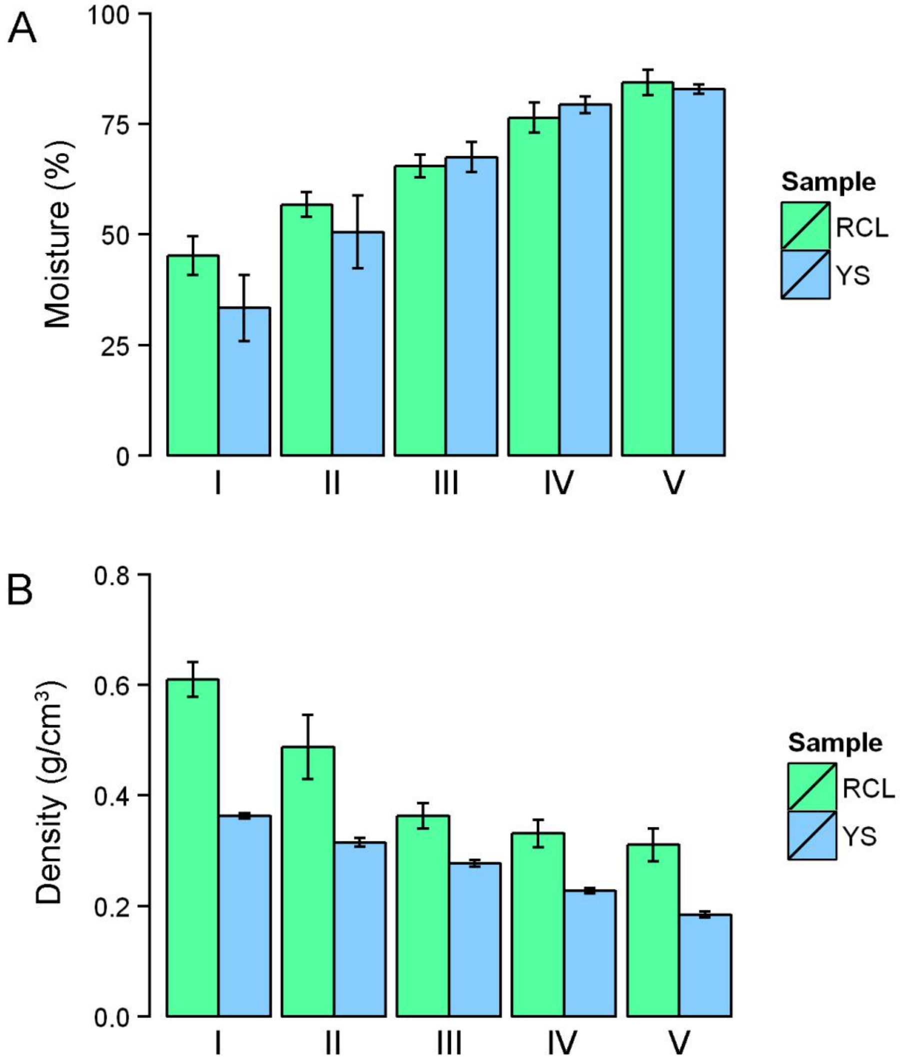

S2. Moisture and density of fallen woods of *Q. aliena* var. *acuteserrata* and *P. tabulaeformis* at different decomposition stages.

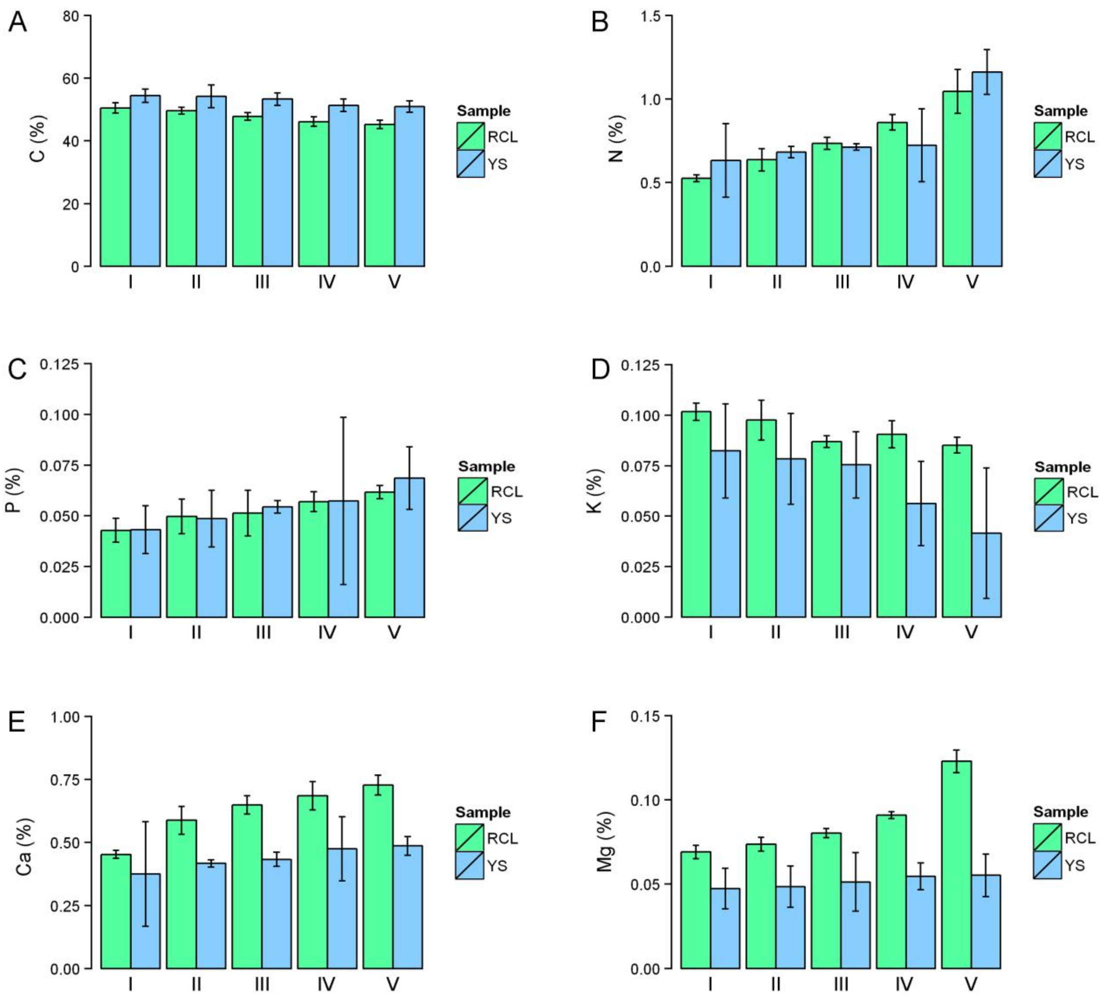

S3. Chemical characteristics for fallen woods of *Q. aliena* var. *acuteserrata* and *P. tabulaeformis* at five decomposition stages.

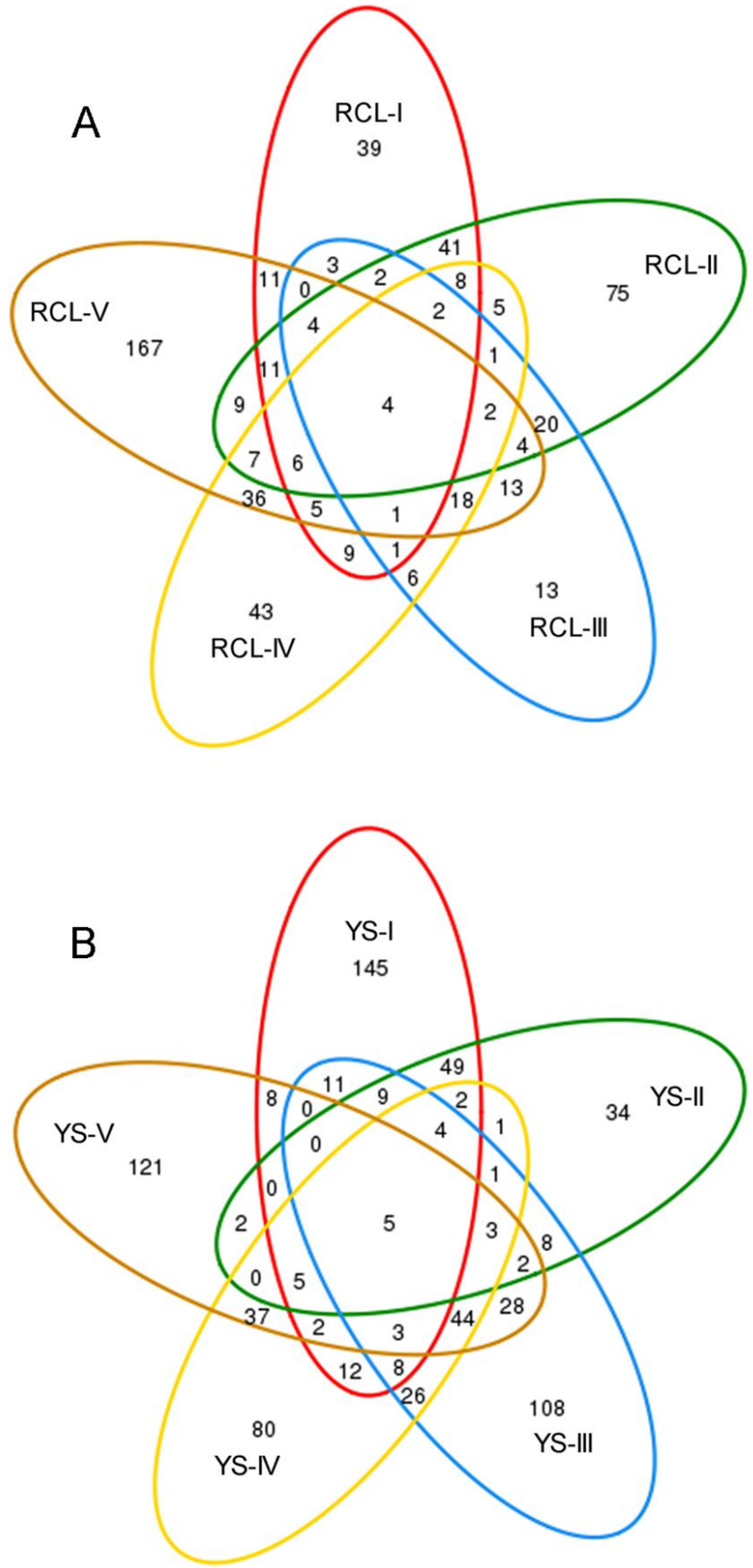

S4. Number patterns of specific and shared fungal OTUs in fallen woods of *Q. aliena* var. *acuteserrata* (A) and *P. tabulaeformis* (B) at different decomposition stages.
